# Supplementary material for: Assessing awareness of blood cancer symptoms and barriers to symptomatic presentation: measure development and results from a population survey in the UK
Source: BMC Cancer. 2023 Jul 6;23:633. doi: 10.1186/s12885-023-11149-x (PMC10324260; doi:10.1186/s12885-023-11149-x)
Supplement: Supplementary file 1 — Supplementary Material 1 [file 12885_2023_11149_MOESM1_ESM.docx]

**Appendices:**

**Table 1: Test re-test for blood cancer awareness**

| Signs or symptoms | Correctly identified T1 | Correctly identified T2 | ICC | 95%CI |
| --- | --- | --- | --- | --- |
|  | % (N) | % (N) |  |  |
| Weight loss | 67.55 (204) | 72.52 (72.52) | 0.58 | 0.48-0.67 |
| Bruising | 64.9 (196) | 69.54 (69.54) | 0.72 | 0.65-0.78 |
| Bleeding | 66.89 (202) | 72.19 (218) | 0.60 | 0.49-0.68 |
| Lumps/swell | 62.91 (190) | 63.58 (192) | 0.51 | 0.39-0.61 |
| Breath | 40.4 (122) | 44.04 (133) | 0.60 | 0.49-0.68 |
| Cough | 28.81 (87) | 22.19 (67) | 0.55 | 0.43-0.64 |
| Night sweats | 29.47 (89) | 36.75 (111) | 0.59 | 0.49-0.68 |
| Infections | 51.32 (155) | 52.98 (160) | 0.58 | 0.48-0.67 |
| Fever | 45.7 (138) | 47.35 (143) | 0.59 | 0.49-0.68 |
| Rash/itchy skin | 54.97 (166) | 54.64 (165) | 0.54 | 0.42-0.63 |
| Pain (bones/joints) | 48.68 (147) | 50.66 (153) | 0.49 | 0.36-0.59 |
| Pain (stomach) | 51.32 (155) | 50.66 (153) | 0.52 | 0.40-0.62 |
| Fatigue | 61.59 (186) | 64.57 (64.57) | 0.57 | 0.46-0.66 |
| Pallor | 58.94 (178) | 68.21 (206) | 0.62 | 0.52-0.70 |
|  | Mean (SD)  *Median (IQR)* | Mean (SD)  *Median (IQR)* |  |  |
| Total symptom awareness^1^ | 7.24 (4.04)  *8 (4, 13)* | 7.61 (4.07)  *8 (4, 13)* | 0.74 | 0.68 - 0.79 |

1 For sub-set that answered at both timepoints

**Table 2: Descriptive statistics and internal reliability for re-consultation, body vigilance and enablement**

|  |  |  |  | **IQR** | |  |  |  |
| --- | --- | --- | --- | --- | --- | --- | --- | --- |
| **Items** | **Mean** | **SD** | **Median** | **25%** | **75%** | **Item-test correlation** | **Covariance** | **Item α** |
| **Candidacy**^1^  *Comfortable going back to my GP with the same symptom/health problem…* | | | | | | | | |
| If got worse or didn’t go away | 4.15 | 0.85 | 4 | 4 | 5 | 0.59 | 0.44 | 0.75 |
| After a test result suggested there was nothing to worry about, but symptoms persisted | 3.80 | 0.83 | 4 | 3 | 4 | 0.62 | 0.41 | 0.71 |
| To request further tests, scans or investigations | 3.88 | 0.88 | 4 | 3 | 5 | 0.67 | 0.35 | 0.66 |
| *Scale* |  |  |  |  |  |  |  |  |
| Cronbach’s α |  |  |  |  |  |  | 0.40 | 0.79 |
| McDonald’s Ω |  |  |  |  |  |  |  | 0.79 |
| **Body vigilance**  *Thinking about how you view changes in your body* | | | | | | | | |
| I am very aware of changes in my body | 4.03 | 0.80 | 4 | 4 | 5 | 0.88 | 0.39 | 0.72 |
| I pay close attention to changes in my body | 3.88 | 0.85 | 4 | 3 | 4 | 0.87 | 0.38 | 0.74 |
| I just know when something isn’t right | 3.99 | 0.81 | 4 | 4 | 5 | 0.84 | 0.44 | 0.79 |
| **Scale** |  |  |  |  |  |  |  |  |
| Α |  |  |  |  |  |  | 0.41 | 0.82 |
| McDonald’s Ω |  |  |  |  |  |  |  | 0.82 |
| **Enablement**^2^  *Last time visited HCP at GP practice about symptom/health problem felt…* | | | | | | | | |
| Able to understand symptom/health problem | 2.75 | 0.85 | 3 | 2 | 3 | 0.82 | 0.47 | 0.89 |
| Able to cope with symptom/health problem | 2.56 | 0.79 | 3 | 2 | 3 | 0.83 | 0.48 | 0.89 |
| Able to keep yourself health | 2.56 | 0.82 | 2 | 2 | 3 | 0.87 | 0.45 | 0.88 |
| Confident about your health | 2.41 | 0.84 | 2 | 2 | 3 | 0.86 | 0.45 | 0.88 |
| Able to help yourself | 2.58 | 0.88 | 2 | 2 | 3 | 0.88 | 0.43 | 0.87 |
| **Scale** |  |  |  |  |  |  |  |  |
| Cronbach’s α |  |  |  |  |  |  | 0.46 | 0.90 |
| McDonald’s Ω |  |  |  |  |  |  |  | 0.92 |

1 Rated on a 5-point scale from Strongly agree to strongly disagree. Higher scores indicate higher candidacy

2 Rated on a 4-point scale from Less to Much Better. Higher scores indicate higher enablement

**Table 3:** **Test re-test for re-consultation**

|  | ICC | 95% CI |
| --- | --- | --- |
| *Comfortable going back to my GP with the same symptom/health problem* | | |
| If got worse or didn’t go away | 0.54 | 0.42-0.63 |
| After a test result suggested there was nothing to worry about, but symptoms persisted | 0.54 | 0.43-0.64 |
| To request further test, scans or investigations | 0.50 | 0.37-0.60 |
| Total | 0.61 | 0.52-0.70 |

**Table 4: Descriptive statistics and internal reliability of barriers item and scale**

|  |  |  |  | **IQR** | |  |  |  |
| --- | --- | --- | --- | --- | --- | --- | --- | --- |
| **Items^1^** | **Mean** | **SD** | **Median** | **25%** | **75%** | **Item-test correlation** | **Covariance** | **Item α** |
| Embarrassed talking about symptoms | 2.76 | 1.43 | 2 | 2 | 4 | 0.79 | 0.47 | 0.89 |
| Worried what they might find wrong | 3.25 | 1.12 | 3 | 2 | 4 | 0.60 | 0.51 | 0.90 |
| Worry not take symptoms seriously | 3.13 | 1.29 | 3 | 2 | 4 | 0.73 | 0.49 | 0.89 |
| Worry about treatment | 3.09 | 1.22 | 3 | 2 | 4 | 0.69 | 0.50 | 0.90 |
| HCP doesn’t explain in way I understand | 2.85 | 1.25 | 3 | 2 | 4 | 0.74 | 0.49 | 0.89 |
| Nothing puts me off | 2.43 | 1.09 | 2 | 2 | 3 | 0.20 | 0.56 | 0.91 |
| Easy to explain symptoms | 2.17 | 0.99 | 2 | 1 | 3 | 0.15 | 0.56 | 0.91 |
| Wasting HCP time | 3.20 | 1.34 | 3 | 2 | 4 | 0.72 | 0.49 | 0.89 |
| Someone who makes a fuss | 3.33 | 1.17 | 4 | 3 | 4 | 0.58 | 0.51 | 0.90 |
| Difficult to see specific HCP | 3.56 | 1.23 | 4 | 3 | 5 | 0.57 | 0.51 | 0.90 |
| Talk to receptionist about symptoms | 3.53 | 1.10 | 4 | 3 | 4 | 0.49 | 0.53 | 0.90 |
| Putting strain on NHS | 3.20 | 1.21 | 3 | 2 | 4 | 0.61 | 0.51 | 0.90 |
| Appointment at convenient time | 3.55 | 1.28 | 4 | 2 | 5 | 0.62 | 0.50 | 0.90 |
| Too busy | 2.62 | 1.29 | 2 | 2 | 4 | 0.72 | 0.49 | 0.89 |
| Impact on employment from taking time off | 2.59 | 1.29 | 3 | 1 | 4 | 0.65 | 0.50 | 0.90 |
| Difficult to discuss remotely | 3.29 | 1.26 | 3 | 2 | 4 | 0.62 | 0.51 | 0.90 |
| Many things to worry about | 2.82 | 1.18 | 3 | 2 | 4 | 0.73 | 0.50 | 0.89 |
| Thought related to existing condition/illness | 3.13 | 1.14 | 3 | 2 | 4 | 0.66 | 0.51 | 0.90 |
| **Scale** |  |  |  |  |  |  |  |  |
| Cronbach’s α |  |  |  |  |  |  | 0.51 | 0.90 |
| McDonald’s Ω |  |  |  |  |  |  |  | 0.90 |

1 Rated on a 5-point scale from Strongly agree to strongly disagree. Higher scores indicate higher barriers

**Table 5: Test re-test for barriers to help-seeking**

| Barrier item | ICC | 95% CI lower | upper |
| --- | --- | --- | --- |
| I found it embarrassing talking about my symptoms | 0.79 | 0.74 | 0.83 |
| I worried what they might find wrong with me | 0.71 | 0.64 | 0.77 |
| I worried they wouldn’t take my symptom(s) seriously | 0.77 | 0.71 | 0.82 |
| I worried about the possibility of having treatment | 0.70 | 0.62 | 0.76 |
| The healthcare professional doesn't explain things in a way I can understand | 0.73 | 0.66 | 0.78 |
| Nothing put me off/ delayed me in seeking medical attention | 0.60 | 0.49 | 0.68 |
| I felt I could easily explain/ talk about my symptom(s) | 0.55 | 0.44 | 0.64 |
| I worried about wasting the healthcare professional’s time | 0.80 | 0.75 | 0.84 |
| I didn’t want to be seen as someone who makes a fuss | 0.75 | 0.68 | 0.80 |
| I found it difficult to get an appointment with a specific health professional at the GP practice (e.g. doctor) | 0.74 | 0.68 | 0.79 |
| I didn’t want to talk to a receptionist/administrative person about my symptom(s) | 0.72 | 0.64 | 0.77 |
| I worried about putting extra strain on the NHS | 0.76 | 0.70 | 0.81 |
| I found it difficult to get an appointment at a convenient time | 0.84 | 0.80 | 0.87 |
| I was too busy to make time to seek medical attention | 0.78 | 0.72 | 0.82 |
| I worried about the impact on my employment/ work from taking time off | 0.79 | 0.73 | 0.83 |
| It would have been difficult for me to discuss my health problem remotely (by phone, email or video call) | 0.65 | 0.56 | 0.72 |
| I had too many other things to worry about | 0.75 | 0.69 | 0.80 |
| I thought the symptom was related to an existing illness or condition | 0.68 | 0.60 | 0.74 |
| Total | 089 | 0.87 | 0.92 |

**Table 6: Eigenvalues, percentage of variance and cumulative percentage for the four-factor barrier scale**

| Factor | Eigenvalue | Percentage of variance | Cumulative percentage of variance |
| --- | --- | --- | --- |
|  |  |  |  |
| Factor1 | 7.20 | 40.0 | 40.0 |
| Factor2 | 1.72 | 9.6 | 49.6 |
| Factor3 | 1.56 | 8.7 | 58.3 |
| Factor4 | 1.21 | 6.7 | 65.0 |

**Table 7: Correlations among four factors**

|  | Factor1 | Factor2 | Factor3 | Factor4 |
| --- | --- | --- | --- | --- |
| Factor1 | - |  |  |  |
| Factor2 | 0.52 | - |  |  |
| Factor3 | 0.46 | 0.48 | - |  |
| Factor4 | 0.13 | 0.02 | 0.11 | - |

| **Table 8 Factor loadings and uniqueness for oblique rotation four-factor solution for the 18 barrier items** | | | | | |
| --- | --- | --- | --- | --- | --- |
| Barrier items | Factor loading | | | |  |
|  | Factor1 | Factor2 | Factor3 | Factor4 | Unique-ness |
| I found it embarrassing talking about my symptoms | **0.46** | 0.41 | 0.05 | 0.11 | 0.34 |
| I worried what they might find wrong with me | **0.74** | -0.13 | 0.10 | 0.11 | 0.43 |
| I worried they wouldn’t take my symptom(s) seriously | **0.41** | 0.11 | 0.37 | 0.14 | 0.41 |
| I worried about the possibility of having treatment | **0.69** | 0.01 | 0.11 | 0.15 | 0.38 |
| I worried about wasting the healthcare professional’s time | **0.76** | 0.24 | -0.13 | -0.10 | 0.29 |
| I didn’t want to be seen as someone who makes a fuss | **0.83** | 0.01 | -0.17 | -0.05 | 0.41 |
| I worried about putting extra strain on the NHS | **0.74** | 0.11 | -0.07 | -0.23 | 0.40 |
| I was too busy to make time to seek medical attention | 0.07 | **0.86** | -0.05 | -0.02 | 0.24 |
| I worried about the impact on my employment/ work from taking time off | -0.03 | **0.87** | -0.05 | -0.01 | 0.31 |
| I had too many other things to worry about | 0.13 | **0.77** | -0.03 | 0.11 | 0.30 |
| I thought the symptom was related to an existing illness or condition | 0.03 | **0.65** | 0.18 | -0.10 | 0.41 |
| The healthcare professional doesn't explain things in a way I can understand | 0.25 | 0.33 | **0.38** | -0.02 | 0.40 |
| I found it difficult to get an appointment with a specific health professional at the GP practice (e.g. doctor) | -0.11 | 0.01 | **0.89** | -0.06 | 0.28 |
| I didn’t want to talk to a receptionist/administrative person about my symptom(s) | 0.23 | -0.33 | **0.77** | -0.06 | 0.41 |
| I found it difficult to get an appointment at a convenient time | -0.21 | 0.23 | **0.81** | 0.02 | 0.28 |
| It would have been difficult for me to discuss my health problem remotely (by phone, email or video call) | 0.01 | 0.39 | **0.40** | -0.03 | 0.53 |
| Nothing put me off/ delayed me in seeking medical attention | -0.02 | 0.04 | -0.05 | **0.87** | 0.25 |
| I felt I could easily explain/ talk about my symptom(s) | -0.03 | -0.02 | -0.03 | **0.88** | 0.23 |
